# Chemical Ecology of Parasitic Hymenoptera

**DOI:** 10.1155/2016/4298150

**Published:** 2016-02-22

**Authors:** Giovanni Benelli, Kent M. Daane, Roxina Soler, Johannes Stökl

**Affiliations:** ^1^Department of Agriculture, Food and Environment, University of Pisa, Via del Borghetto 80, 56124 Pisa, Italy; ^2^Department of Environmental Science, Policy and Management, University of California, Berkeley, CA 94720-3114, USA; ^3^Department of Terrestrial Ecology, Netherlands Institute of Ecology (NIOO-KNAW), Droevendaalsesteeg 10, 6708 PB Wageningen, Netherlands; ^4^Institute for Zoology, University of Regensburg, Universitätsstraße 31, 93053 Regensburg, Germany

Over the past one hundred years, the evolutionary chemical ecology of arthropods has been deeply investigated by a wide range of scientists, including chemists, ecologists, neurobiologists, entomologists, and behavioural and evolutionary biologists. Among insects, a substantial number of studies focused on the ecology of parasitic Hymenoptera, particularly wasp species that have evolved to utilize insect herbivores. Parasitoids are therefore considered key organisms in both natural and agricultural systems, for their role in ecosystem services and biological pest control. These fascinating animals rely on a range of communication channels during their life, including visual, auditory, and olfactory channels.

A full understanding of the chemical ecology and evolution of Hymenoptera parasitoids can help develop novel or improve current biological control programs. At an operational level, behavioural knowledge would help to improve mass-rearing techniques, evaluate release rates in a given habitat, and predict parasitoid effectiveness against a variety of hosts. Although extensive research has been carried out on these topics in recent years, there are still significant gaps in our knowledge of the chemical ecology of many parasitic wasps. In this scenario, this special issue presents review and original research papers covering different facets of the chemical ecology of parasitic wasps.

The effects of abiotic factors on biotic interactions mediated by herbivore-induced plant volatiles have received only limited attention. In the review article “Effects of Abiotic Factors on HIPV-Mediated Interactions between Plants and Parasitoids,” C. Becker et al. highlighted that HIPV can be influenced by the plant growing conditions, which could have major implications for pest management. Indeed, quantitative and qualitative changes in HIPV blends can improve or impair biocontrol. Enhanced emission of HIPV may attract a larger number of natural enemies. Reduced emission rates or altered compositions, however, may render blends imperceptible to parasitoids and predators. Predicting the outcome of these changes is important for food production and for ecosystems affected by global climate change.

Even though Braconidae wasps are employed as biological control agents against Tephritidae flies, their use is still limited in olive groves against the olive fruit fly, due to low parasitisation efficiency. Besides visual cues, olfactory stimuli can provide key information driving parasitoid host location processes. In “VOCs-Mediated Location of Olive Fly Larvae by the Braconid Parasitoid* Psyttalia concolor*: A Multivariate Comparison among VOC Bouquets from Three Olive Cultivars,” G. Giunti et al. investigated the effect of* Bactrocera oleae* infestation on olive fruits of different cultivars. Particular emphasis was given to the impact of infestation on volatile organic compound emissions, in order to evaluate the occurrence of putative HIPV, which may be useful to enhance the parasitoid efficacy in control programs against the olive fruit fly.

In “Electrophysiological and Behavioral Responses of* Theocolax elegans* (Westwood) (Hymenoptera: Pteromalidae) to Cereal Grain Volatiles,” G. Germinara et al. studied the olfactory responses of* T. elegans*, a pteromalid wasp that parasitises immature stages of stored-product insect pests, to volatiles emitted by healthy wheat grains, their hexane extracts, and different doses of three individual compounds previously identified in cereal grain odours. In Y-tube olfactometer bioassays, odours from healthy wheat grains and their hexane extracts were attractive to both sexes of* T. elegans*. Hexane extracts elicited arresting effects in Petri dish arena. The three synthetic compounds valeraldehyde, maltol, and vanillin elicited dose-dependent responses in both male and female adult wasps confirming the capability of the peripheral olfactory systems to perceive cereal volatiles. In behavioural bioassays, different doses of vanillin were significantly attractive to both sexes.

The cues routing the host-searching behaviour of Mymaridae wasps have been scarcely studied. In the research article “*Anagrus breviphragma* Soyka Short Distance Search Stimuli,” A. Berzolla et al. pointed out that chemicals soluble in polar solvents are more important than host-borne physical cues, when* Anagrus breviphragma* females search for* Cicadella viridis* eggs. Notably, the stimuli that elicit probing and oviposition are not subjected to learning.

Furthermore, two original research articles present information on the identity and behavioural role of some compounds involved in the courtship and mating behaviour of two parasitoids of economic importance. The study “Sexy Mouth Odour? Male Oral Gland Pheromone in the Grain Beetle Parasitoid* Lariophagus distinguendus* (Förster) (Hymenoptera: Pteromalidae)” by K. König et al. analysed the courtship and mating behaviour of* L. distinguendus* from two different lineages, which are sexually isolated because males fail to elicit receptivity in foreign females. They showed that in* L. distinguendus* a nonvolatile male oral pheromone is essential to release the female receptivity signal. In contrast, male wing fanning and antennal contact play a minor role. Additionally, the composition of the oral pheromone depends on the developmental host and females learn the composition upon emergence from the host substrate. In the study “Species Specificity of the Putative Male Antennal Aphrodisiac Pheromone in* Leptopilina heterotoma*,* Leptopilina boulardi*, and* Leptopilina victoriae,*” I. Weiss et al. focus on male antennal aphrodisiac pheromones eliciting female receptiveness in* Leptopilina* parasitoids. They studied the species specificity of the putative male aphrodisiac pheromone of* L. heterotoma, L. boulardi*, and* L. victoriae*. Males elicited receptiveness only in conspecific females, never in the manipulated heterospecific females. Chemical analyses showed the presence of species-specific unsaturated hydrocarbons on the antennae of males. Only trace amounts of these hydrocarbons were found on the antennae of females. These results are of importance for a full understanding and identification of antennal pheromones in parasitic wasps.

Overall, we are grateful to this journal for hosting this special issue, and we hope that the knowledge presented here will be helpful to researchers in chemoecology, behavioural ecology, and biological control, to boost ecofriendly pest management strategies.

*Giovanni Benelli* *Kent M. Daane* *Roxina Soler* *Johannes Stökl*

